# Examining the Feasibility of Clinical Grade CD271^+^ Enrichment of Mesenchymal Stromal Cells for Bone Regeneration

**DOI:** 10.1371/journal.pone.0117855

**Published:** 2015-03-11

**Authors:** Richard J. Cuthbert, Peter V. Giannoudis, Xiao N. Wang, Lindsay Nicholson, David Pawson, Anatole Lubenko, Hiang B. Tan, Anne Dickinson, Dennis McGonagle, Elena Jones

**Affiliations:** 1 Leeds Institute of Rheumatic and Musculoskeletal Medicine, University of Leeds, Leeds, United Kingdom; 2 Institute of Cellular Medicine, University of Newcastle upon Tyne, Newcastle, United Kingdom; 3 National Health Service Blood and Transplant, Leeds Blood Centre, Leeds, United Kingdom; Josep Carreras Leukaemia Research Institute, University of Barcelona, SPAIN

## Abstract

**Introduction:**

Current clinical trials utilize mesenchymal stromal cells (MSCs) expanded in culture, however these interventions carry considerable costs and concerns pertaining to culture-induced losses of potency. This study assessed the feasibility of new clinical-grade technology to obtain uncultured MSC isolates from three human intra-osseous tissue sources based on immunomagnetic selection for CD271-positive cells.

**Materials and Methods:**

MSCs were isolated from bone marrow (BM) aspirates or surgical waste materials; enzymatically digested femoral heads (FHs) and reamer irrigator aspirator (RIA) waste fluids. Flow cytometry for the CD45^−/low^CD73^+^CD271^+^ phenotype was used to evaluate uncultured MSCs before and after selection, and to measure MSC enrichment in parallel to colony forming-unit fibroblast assay. Trilineage differentiation assays and quantitative polymerase chain-reaction for key transcripts involved in bone regeneration was used to assess the functional utility of isolated cells for bone repair.

**Results:**

Uncultured CD45^−/low^CD271^+^ MSCs uniformly expressed CD73, CD90 and CD105 but showed variable expression of MSCA-1 and SUSD2 (BM>RIA>FH). MSCs were enriched over 150-fold from BM aspirates and RIA fluids, whereas the highest MSC purities were obtained from FH digests. Enriched fractions expressed increased levels of BMP-2, COL1A2, VEGFC, SPARC and CXCL12 transcripts (BM>RIA>FH), with the highest up-regulation detected for CXCL12 in BM (>1300-fold). Following culture expansion, CD271-selected MSCS were tri-potential and phenotypically identical to plastic adherence-selected MSCs.

**Discussion:**

A CD271-based GMP-compliant immunomagnetic selection resulted in a substantial increase in MSC purity and elevated expression of transcripts involved in bone formation, vascularisation and chemo-attraction. Although this technology, particularly from RIA fluids, can be immediately applied by orthopaedic surgeons as autologous therapy, further improvements in MSC purities and pre-clinical testing of product safety would be required to develop this process for allogeneic applications.

## Introduction

Culture expanded mesenchymal stromal cells (MSCs), also designated mesenchymal stem cells, have undergone trials as therapeutic agents to treat a range of conditions including osteogenesis imperfecta [1], cartilage defects [2], acute myocardial infarction [3] and steroid resistant graft versus host disease [4]. Thus far, expanded MSC therapy trials have resulted in variable clinical outcomes, even for the same disease. This may be related to the MSC source or route of administration or to differences in culture conditions and the degree of expansion employed [5–8]. Besides additional safety concerns associated with prolonged ex vivo cell cultivation including the risk of transformation [9–11], therapies utilising expanded cells must fulfil good manufacturing practice (GMP) conditions which makes the costs of therapies prohibitively high [12].

Conversely, uncultured cells, including CD34 selected or CD3, CD19 depleted cellular products following CliniMACS cell selection, have been used for haematopoietic stem cell transplantation for decades and have revolutionized the treatment of haematological diseases and saved the lives of thousands of patients [13]. There is therefore a strong impetus to develop similar clinical-grade procedures for the isolation of uncultured MSCs, especially given the potency and differentiation potentials that such MSCs may possess [14].

Although there have been attempts to increase MSC purity by physical means [15], positive selection based on a specific MSC marker offers an appealing alternative. Because MSCs are very rare in BM aspirates [16–19], the isolation of pure uncultured BM MSCs in the ‘research-scale’ settings is best achieved by cell sorting with a combination of positive and negative markers [17,20–23]. Amongst a number of positive markers proposed in the past [24–26], CD271 offers unique selectivity, defined as the least cross-reactivity with contaminating haematopoietic-lineage cells [7,17,23,27,28]. This is highly advantageous for the establishment of a single marker-based, immunomagnetic enrichment procedure.

Femoral Heads (FH) removed as part of total hip replacement surgery, represent a potential MSC source that is currently discarded but could be used in autologous settings to strengthen implant integration with the remaining bone [29,30]. We and others have previously demonstrated that enzymatic digestion of FH bone releases large numbers of MSCs that have a similar phenotype to MSCs in BM aspirates [31,32]. Reamer irrigator aspirator (RIA) waste fluid is another surgical waste by-product that is rich in MSCs, which is currently discarded [33–35]. It is a particularly attractive source of MSCs since unlike FHs, it does not require enzymatic digestion and can be used in autologous settings in complex bone reconstruction procedures.

This study therefore investigated the potential of clinical-grade anti-CD271 microbeads, in combination with the CliniMACS device, to isolate human uncultured MSCs from BM aspirates. Our secondary aim was to investigate whether this technology could be used to enrich MSCs from other intra-osseous sources, namely the FH digests and RIA waste fluid [32,36], both waste products of surgical interventions.

## Materials and Methods

### Ethics statement

All patients gave informed written consent and research was carried out in compliance with the Helsinki Declaration. Ethics committee approval for this study was obtained from the local National Health Service Research & Development Department, National Research Ethics Service, Leeds East and West Research Ethics Committees under permit numbers 06/Q1206/127 and 07/Q1205/27 respectively.

### Patient cohorts

BM aspirates were obtained from patients undergoing elective orthopaedic surgery or were surplus BM donated by healthy donors, for haematopoietic stem cell transplantation (n = 23, median age 38, range: 4–72). FHs were collected from patients with osteoarthritis admitted for total hip arthroplasty (n = 6, median age 70, range: 58–81). RIA waste fluid (mean volume 867ml) were obtained from patients admitted for the treatment of fracture non-union involving autologous bone grafting (n = 6, median age 38, range: 28–63).

### CD271 positive selection using QuadroMACS for functional characterisation

Mononuclear cells (MNCs) isolated from BM aspirates, FHs and RIA waste fluid using Lymphoprep reagent (Axis-Shield, Oslo, Norway) were labelled with clinical grade anti-CD271 microbeads (Miltenyi Biotec GmbH, Germany), according to the manufacturer’s instructions. These consist of murine anti-CD271 monoclonal antibodies (clone information confidential) conjugated to superparamagnetic iron dextran particles. Once labelled, cells were separated into positive and negative fractions using the QuadroMACS system (Miltenyi Biotec). Cells from the positive fraction were seeded into a T25 flask (Corning, NY, USA) and cultured in GMP compliant MSC expansion medium MSCGM Bulletkit (Lonza, Berkshire, UK). Three days later, culture medium was replaced and subsequently changed twice weekly. Once the cells (now denoted CD271-MSC) reached 80% confluence, the adherent cells were harvested with 0.25% trypsin/1mM ethylenediaminetetraacetic acid (EDTA) solution (Sigma-Aldrich, Dorset, UK) and passaged at the seeding density of 4x10^3^/cm^2^ for further expansion. Control plastic adherent MSCs (PA-MSC) were initiated according to standard protocols [36] and passaged using the same seeding density as CD271-MSCs. At each passage cell population doubling (PD) time was calculated.

To investigate MSC chondrogenic, osteogenic and adipogenic potentials, cells were centrifuged in chondrogenic pellets (2x10^5^cells/pellet) or seeded into 6-well plates (osteogenic and adipogenic seeding density of 2x10^4^/well and 2x10^5^/well, respectively). Cells were cultured in either StemMACS ChondroDiff Media, StemMACS OsteoDiff Media or StemMACS AdipoDiff Media (all Miltenyi Biotec) with twice-weekly media changes. After 21 days of differentiation, chondrogenic pellets were fixed in 10% formalin, embedded in paraffin and 5μm sections were stained with 0.1% Alcian Blue to detect proteoglycan deposition. Osteogenic cultures were first stained with alkaline phosphatase substrate solution followed by a 1-hour treatment with 3% silver nitrate to detect mineralization. Adipogenic cultures were fixed in 60% isopropanol before being stained with Oil Red O solution. Images were captured using a Zeiss Axio Imager II (Carl Zeiss) and processed using NIS Elements Imaging Software version 4.0 (Nikon).

### Cell phenotyping

Phenotypic analysis of cells from BM, FH and RIA was carried out using flow cytometry. Samples were incubated with antibodies or isotype controls in FACS buffer (PBS pH7.4, 1% bovine serum albumin) according to the manufacturer’s instructions. In each case 4,6-diamidino-2-phenylindole (DAPI, Sigma-Aldrich) or aqua dead cell stain (Life technologies, Paisley, UK) was added prior to acquisition to exclude dead cells.

Previously selected, expanded PA-MSCs and CD271-MSCs from each passage were probed for expression of CD73, CD105, CD90, CD34, CD45, CD19, CD14 and HLA-DR (Table 1) [37]; this data was acquired using a BD FACSCanto II flow cytometer (BD). All data was analysed using FlowJo v7.6.5 software (Treestar, Oregon, USA).

**Table 1:** Antibodies used.

Specificity	Conjugate	Clone	Manufacturer
**Cultured MSC phenotype**			
**CD14**	**FITC**	**MφP9**	**BD**
**CD19**	**FITC**	**HIB19**	**BD**
**CD34**	**FITC**	**581**	**BD**
**CD45**	**FITC**	**2D1**	**BD**
**CD73**	**PE**	**AD2**	**BD**
**CD90**	**PerCP Cy5.5**	**5E10**	**BD**
**CD105**	**APC**	**266**	**BD**
**HLA-DR**	**APC-H7**	**L243**	**BD**
**Extended phenotyping**			
**CD3**	**V450**	**UCHT1**	**BD**
**CD14**	**PE**	**M5E2**	**BD**
**CD19**	**Alexa fluor 700**	**HIB19**	**BD**
**CD31**	**BV610**	**WM59**	**Biolegend**
**CD34**	**APC**	**8G12**	**BD**
**CD45**	**PEcy7**	**HI30**	**BD**
**CD45**	**BV780**	**H130**	**Biolegend**
**CD73**	**PerCP Cy5.5**	**AD2**	**Mitenyi Biotec**
**CD90**	**PECy7**	**5E10**	**BD**
**CD105**	**FITC**	**43A4E1**	**Mitenyi Biotec**
**CD146**	**FITC**	**3A6**	**BD**
**CD271**	**APC**	**ME20.4-1H4**	**Mitenyi Biotec**
**CD271**	**PE Vio770**	**ME20.4-1H4**	**Mitenyi Biotec**
**CD235a**	**PE**	**GA-R2**	**BD**
**MSCA-1**	**PE**	**W8B2**	**Mitenyi Biotec**
**SUSD2**	**APC**	**W5C5**	**Biolegend**
**Assessment of MSC content**			
**CD45**	**PEcy7**	**HI30**	**BD**
**CD73**	**PE**	**AD2**	**BD**
**CD271**	**APC**	**ME20.4-1H4**	**Mitenyi Biotec**

All antibodies used for phenotypic and enrichment analysis. All antibodies listed were monoclonal and raised in mouse.

MSCs identified by negative to low expression of CD45 and positive expression of CD271 (CD45^−/low^CD271^+^) [16,32,34], as well as cells with positive expression of CD45 and low expression of CD271 (CD45^+^CD271^low^) were examined in detail. Expression of a range of cell surface markers was examined by incubation with monoclonal antibodies; CD3, CD14, CD19, CD31, CD34, CD73, CD90, CD105, CD146, CD235a, mesenchymal stem cell antigen 1 (MSCA-1) and sushi domain containing 2 (SUSD2) [23,38,39] (Table 1). Antigen expression on these populations was detected using a BD biosciences LSR II flow cytometer (BD Biosciences, Oxford, UK).

### Processing of BM aspirates, FH specimens and RIA fluids prior to CliniMACS procedures

BM aspirates were harvested as previously described [16], with a minor modification in order to ensure maximal MSC yields [40]. In brief, 5ml of BM was harvested from the first needle insertion site, the needle was subsequently repositioned and a further 5ml was harvested; this was repeated until a total volume of 20ml had been collected. A small volume, 1ml pre-enrichment fraction (Pre) was retained for further analysis. A subset of these samples were taken forward for CliniMACS enrichment, in which case the remaining sample was transferred into a sterile 600ml silicone transfer bag (Miltenyi Biotec). Samples not selected for enrichment were used for direct MSC enumeration by flow cytometry using a CD45/CD271/CD73 antibody combination (Table 1), as previously described [16].

Once removed, FHs were bisected by the operating surgeon and stored immersed in sterile saline overnight at 4°C. The following morning, FHs were removed from saline, broken into small fragments (<1g) using a sterile Noviomagus bone mill (De Puy, Leeds, UK) and digested with 600U/ml collagenase (Worthington, Lakewood, US) for 4 hours at 37°C. The liquid fraction was passed through a 70μm filter (BD) to remove any remaining bone fragments and loaded into a 600ml transfer bag. The RIA waste fluid was directly transferred from the RIA device’s collection bag into two 600ml transfer bags.

### Clinical-grade MSC selection using CD271 microbeads and the CliniMACS system

Prior to separation, cells were washed (400xg for 20 minutes) with GMP-grade PBS/EDTA (Miltenyi Biotec) containing GMP-grade 5% w/v human serum albumin (HSA, Bio Products Laboratory, Elstree, UK) and the supernatant was subsequently discarded using a closed system, in which all sample or waste bags were connected using a TSCDI sterile tube welder and detached using a sterile tube fuser (both from Terumo).

The volume in the transfer bag was next adjusted to 95ml by addition of PBS/EDTA/HSA buffer as above, to which the contents of one vial of anti-CD271 microbeads was added. The cell/bead mixture was incubated for 30 minutes on a tilting shaker at room temperature (RT). Subsequently, a second wash was performed as above followed by volume adjustment to 95ml. The transfer bag was next loaded onto the CliniMACS device and attached to a CliniMACS tubing set. An automated protocol [41] was initiated resulting in separation into a positive (Post) and a negative fractions. The Post fraction was eluted in 40ml of PBS/EDTA/HSA buffer and concentrated by centrifugation to 1ml for further analysis of purity by flow cytometry and CFU-F assay.

CFU-F assays were performed in duplicate as previously described [36]. Pre and Post fractions were seeded at 1x10^5^ and 1x10^4^ nucleated cells/dish, respectively.

### Flow cytometry to assess MSC content before and after selection

For the staining, 50μl of the Pre and Post enrichment fractions originating from BM aspirates, enzymatically digested FH or RIA waste fluid were incubated with CD45, CD271, and CD73 (Table 1) for 15 minutes at RT at manufacturer’s recommended concentrations. Erythrocytes were lysed by the addition of 2ml ammonium chloride solution (168mM NH_2_Cl, 10mM KHCO_3_, 1mM EDTA, pH8.0) containing 0.5μg/ml DAPI (Sigma) and incubation at RT for 10 minutes. MSCs were defined as CD45^-/low^CD271^+^CD73^+^ cells as described previously [16,24,27].

### Quantitative real time PCR

In order to examine the potential enrichment of key transcripts involved in bone regeneration as a result of CD271 selection, RNA was isolated from the total cellular component of BM, FH and RIA samples pre and post CD271 selection. Cells were lysed and RNA isolated using Norgen Total RNA Isolation Kit (Geneflow, Lichfield, UK). Following removal of genomic DNA, cDNA was produced using TaqMan MultiScribe reverse transcription reagents (Life Technologies). TaqMan gene expression assays were then used according to the manufacturer’s instructions to measure transcript expression of genes important for bone regeneration. These were bone morphogenic protein 2 (BMP-2) an essential mediator of fracture healing [42], vascular endothelial growth factor C (VEGFC) an important stimulator of angiogenesis [43], Collagen type I alpha 2 (COL1A2) and osteonectin (SPARC) two factors involved in matrix deposition and mineralisation [34] and Stromal derived factor 1 (CXCL12) a chemokine capable of influencing chemotaxis of a wide range of cell types including MSCs [44,45]. Transcript expression was measured relative to the housekeeping gene hypoxanthine-guanine phosphoribosyltransferase (HPRT) in three matched samples, Pre and Post enrichment, from each tissue.

### Statistics

The Mann-Whitney U test was used for comparison of CD271-MSCs and PA-MSC characteristics. The Spearman rank correlation coefficient was used to assess statistical dependence between CD271 and CD73 expression on uncultured BM MSCs. SPSS version 21 (IBM) was used to calculate all statistics, GraphPad Prism 6 (GraphPad Software) was used to generate all graphs. All bar charts show mean (bar height) and standard deviation.

## Results

### Enrichment of MSCs from BM aspirates using clinical-grade CD271 beads

**Fig 1:**
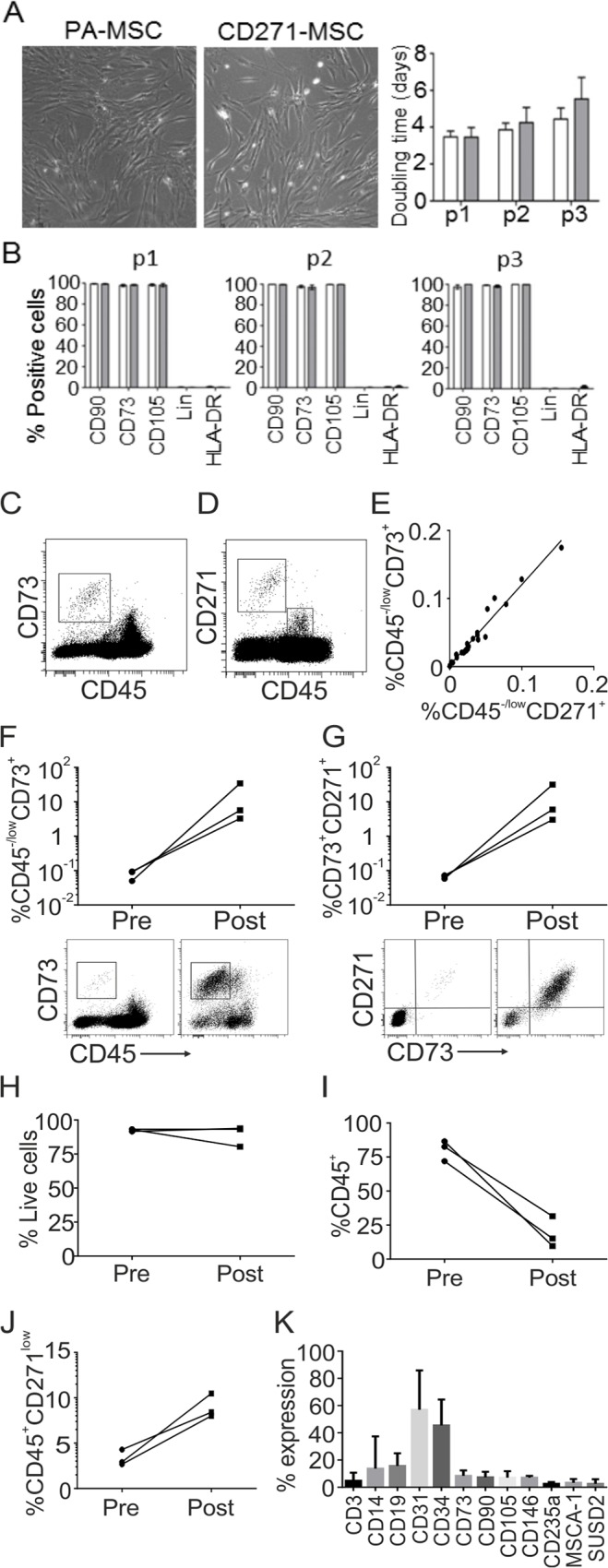
CD271-based enrichment of MSCs from BM aspirates. Comparison of the morphology (x10 magnification) and population doubling time between PA-MSC (open bars) and CD271-MSC isolated using the QuadraMACS system (closed bars, n = 5); Bar charts show means + SEM **(A)**. Immunophenotypic analysis of PA-MSC (open bars n = 5) and CD271-MSC (closed bars) from passage 1 to passage 3. (Lin corresponds to a cocktail of CD14, CD34, CD45 and CD19) **(B)**. Representative dot plots generated from a single BM aspirate showing the CD45^−/low^CD73^+^ population (box) **(C)**, the CD45^−/low^CD271^+^ population (large box) and the CD45^+^CD271^low^ population (small box) **(D)**. The relationship between the proportion of CD45^−/low^CD73^+^ and CD45^−/low^CD271^+^ cells (23 separate BM aspirates collected from 12 donors) **(E)**. The proportion of CD45^−/low^CD73^+^ **(F)** and CD45^−/low^CD73^+^CD271^+^ **(G)** cells, pre and post CliniMACS enrichment with representative dot plots below (n = 3). The proportion of total live cells **(H)** and CD45^+^ leukocytes **(I)** pre and post CliniMACS enrichment. The proportion of CD45^+^CD271^low^ ‘passenger’ cells **(J)**, pre and post enrichment and a detailed analysis of ‘passenger cell’ phenotype **(K)**. (Data F-K, n = 3)

Previous work investigating the functionality of CD271-enriched BM MSCs, in terms of morphology, immunophenotype and tri-lineage potential, has employed research-grade anti-CD271 microbeads [17,24,46]. Here, we assessed the suitability of clinical-grade CD271 microbeads to isolate functionally competent MSCs. CD271-MSC and PA-MSC exhibited similar spindle-shaped fibroblastoid morphology and growth kinetics over the first three passages and showed no significant difference in population doubling times (Fig. 1A). CD271-MSC and PA-MSC also displayed equal positivity for CD73, CD90 and CD105 while lacking expression of the HLA-DR as well as CD14, CD19, CD34 and CD45 (termed Lin) over the first three passages (Fig. 1B), conforming to the classical MSC phenotype [37]. Given that magnetic bead technology relies on binding of microbeads to surface CD271 molecules, there is a possibility that this could compromise further binding of ‘detection’ anti-CD271 antibodies. Furthermore, transient binding of microbeads could also result in internalisation of the CD271 molecule [47]; both factors could therefore affect MSC purity determination post-selection. To account for this, we assessed the value of another MSC-specific molecule: CD73 as an alternative marker for quantification of MSCs before and after CD271-based selections [16,19]. The frequencies of CD45^−/low^CD73^+^ population (Fig. 1C) and the CD45^−/low^CD271^+^ population (Fig. 1D) were measured in parallel in BM aspirates and the correlation between these two populations was examined (Fig. 1E). A remarkably high degree of correlation (R = 0.985, p<0.001) indicated the validity of measuring CD45^−/low^CD73^+^ cells to assess the MSC purity after CD271 selection.

Immunomagnetic enrichment for MSCs using the CliniMACS system and clinical grade CD271-based microbeads was first assessed using BM aspirates (n = 3 donors). Due to volume-related limitations with the starting material (20ml of aspirate collected using an optimized technique), CFU-F assays were not performed in these experiments and MSC purity was assessed by analysis of the CD45^-/low^CD73^+^ cells (Fig. 1F). The proportion of CD45^−/low^CD73^+^ cells before separation (Pre) was 0.078%, consistent with previously published data [19]; it increased to 14.5% following enrichment, thus representing a 186-fold increase (Fig. 1F). To address our concern regarding the binding of ‘detection’ anti-CD271 antibodies to positively-selected cells, the CD271 antibody was also added to the cocktail and the double-positive (CD45^−/low^CD73^+^CD271^+^) population was additionally measured. The proportion of double-positive cells increased from 0.066% to 13.5% representing a 204-fold increase (Fig. 1G). This indicated that microbead binding to the CD271 molecule on the surface of MSCs did not significantly affect binding of the anti-CD271 ‘detection’ antibodies.

Total cell viability was not substantially compromised by the enrichment process (Fig. 1H). CD45^+^ leucocytes [48] represent the majority of cells in BM aspirates. Before enrichment, they represented 80.3% of total cells and although they were substantially depleted following selection, there was still a considerable proportion remaining in the Post fraction (18.7%, Fig. 1I). BM contains a significant portion of cells that express CD271 at a low level as well as CD45 (CD45^+^CD271^low^) [23, 26], illustrated on Fig. 1D, small box. Contamination of the positive fraction with this ‘passenger’ cell type was anticipated. There was however, a relatively modest 15-fold increase in this population from a 0.48% to 7.10% (Fig. 1J). This was quite remarkable since these ‘passenger’ cells are approximately 3-fold more numerous than CD45^−/low^CD271^+^ cells in BM [16]. An extended phenotypic analysis of these ‘passenger’ cells confirmed the lack of expression of classical positive markers of BM MSCs such as CD73, CD90 and CD105. ‘Passenger’ cells also lacked expression of MSCA-1 and SUSD2; whereas, CD34 and CD31 were expressed at variable levels (Fig. 1K). Other contaminating cells present following enrichment included low levels of T-cells (3.8%), B-Cells (3.7%), monocytes (4.5%) and endothelial cells (3.0%).

### Enrichment of MSCs from intra-osseous surgical waste materials using clinical-grade CD271 beads

**Fig 2:**
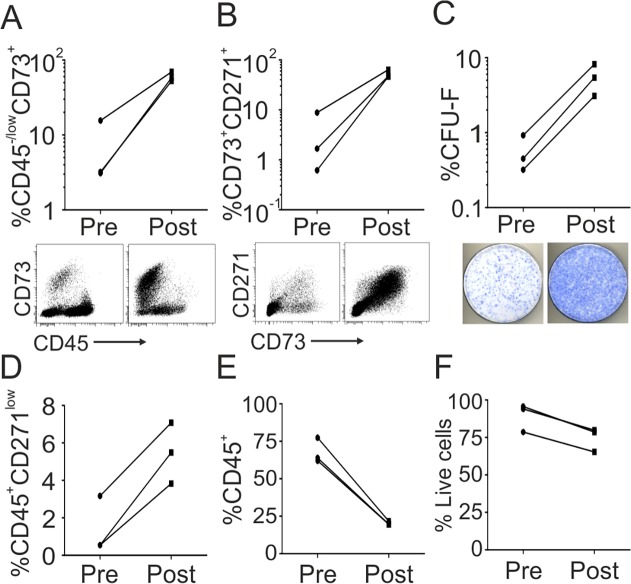
CD271-based enrichment of MSCs from discarded femoral heads. The proportion of CD45^−/low^CD73^+^ **(A)** and CD45^−/low^CD73^+^CD271^+^ **(B)** cells, from enzymatically digested femoral heads pre and post CliniMACS enrichment with representative dot plots below. The proportion of CFU-Fs pre and post CliniMACS enrichment with representative images of CFU-F dishes below **(C)**. The proportion of CD45^+^CD271^low^ ‘passenger’ cells **(D)**, CD45^+^ leukocytes **(E)** and total live cells **(F)** pre and post CliniMACS enrichment. (All data n = 3).

Since obtaining large volumes of high-quality aspirates is problematic, we next evaluated clinical-grade MSC isolation procedures using alternative sources of intra-osseous MSCs. Using cells enzymatically-released from FHs as a starting material, we observed that CD45^−/low^CD73^+^ cells increased by of 8.2-fold from 7.3% in the Pre fraction to 60.0% in the Post fraction (Fig. 2A). The same trends were observed for the double-positive CD45^−/low^CD73^+^CD271^+^ population (14-fold increase, from 3.7% to 52.5%, Fig. 2B). Additional confirmation was provided by CFU-F assay that showed a 9.9-fold increase in the proportion of colony-forming cells (Fig. 2C), giving a mean total 2.4x10^5^ CFU-F in the Post fraction. Similar to our experiments with BM aspirates, a smaller 3.8-fold increase was found in the proportion of CD45^+^CD271^low^ ‘passenger’ cells (from 1.4% to 5.5%, Fig. 2D). Other contaminating cells included T-cells (3.0%), B-cells (5.1%), monocytes (0.7%) and endothelial cells (2.7%). CD45^+^ leucocytes were depleted by 3.3-fold from 67.8% to 20.3% (Fig. 2E). Finally, a slight decrease in cell viability was observed from 89.4% to 74.6% (Fig. 2F), possibly as a result of increased storage, exposure to the enzyme and corresponding processing time.

**Fig 3:**
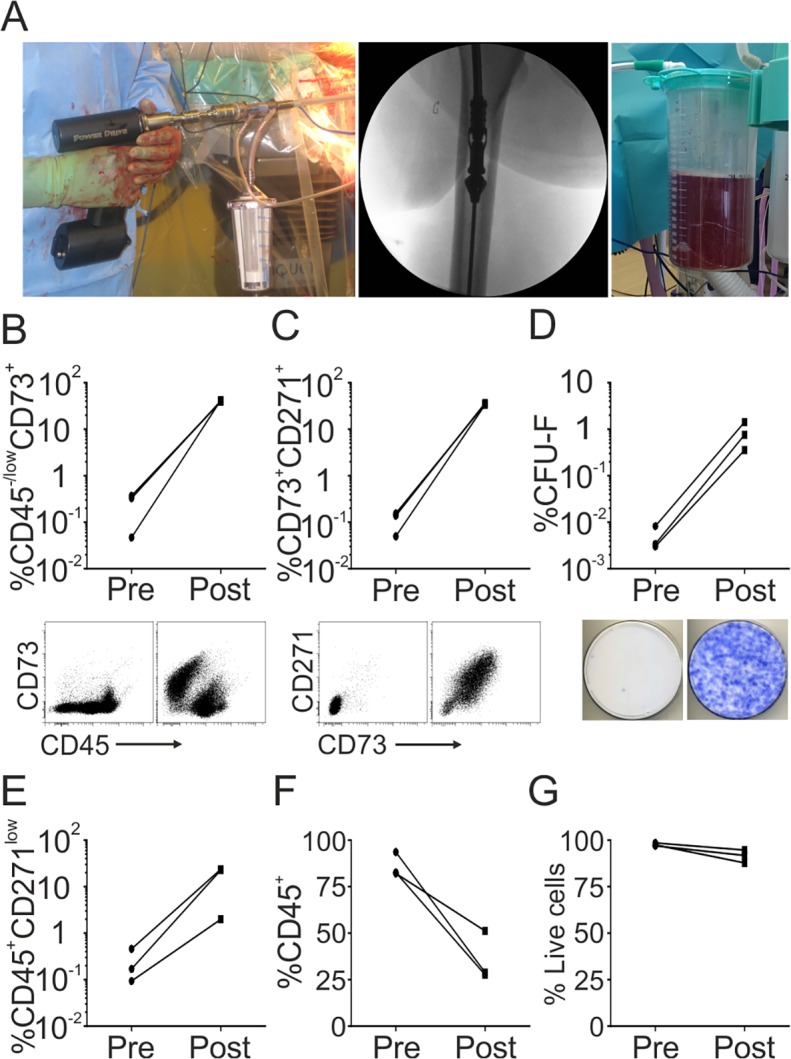
CD271-based enrichment of MSCs from RIA waste fluid. Inter-operative images of the RIA device showing the main body of the device (left panel), the reamer head passing through the intramedullary canal (middle panel) and the waste fluid collection bag (right panel) **(A)**. The proportions of CD45^−/low^CD73^+^ **(B)** and CD45^−/low^CD73^+^CD271^+^ cells **(C)** in RIA waste fluid pre and post CliniMACS enrichment, with representative dot plots shown below. The proportion of CFU-Fs pre and post CliniMACS enrichment and representative CFU-F dishes below **(D)**. The proportion of CD45^+^CD271^low^ ‘passenger’ cells **(E)**, CD45^+^ leukocytes **(F)** and total live cells **(G)** pre and post CliniMACS enrichment. (All data n = 3).

Enrichment was also performed using cells collected in RIA waste fluid, surgical procedure for obtaining RIA waste fluid is shown on Fig. 3A. Following CliniMACS enrichment, CD45^−/low^CD73^+^ cells increased by 163-fold from 0.25% to 40.8% (Fig. 3B); this was supported by measurements of double-positive cells, which increased by 304-fold, from 0.11% to 35.0% (Fig. 3C). CFU-F assay confirmed significant MSC enrichment and showed a 173-fold increase in the proportion of colony-forming cells (Fig. 3D), giving a total of 1.2x10^4^ CFU-Fs in the Post fraction. The number of contaminating cells was low, including T-cells (2.2%), B-cells (4.3%), monocytes (1.5%) and endothelial cells (1.8%). The proportion of CD45^+^CD271^low^ ‘passenger’ cells increased 76-fold, from 0.2% to 18.2% (Fig. 3E) and CD45^+^ leucocytes were depleted by 2.5-fold (Fig. 3F). There was no substantial decrease in cell viability (Fig. 3G).

Altogether these data showed that the best MSC purities and yields were achieved using FH as a starting material, where the initial proportions of MSCs were the highest. RIA waste fluid, on the other hand, gave better cell viability but lower degrees of MSC purity and total MSC number.

### Phenotypic and functional characteristics of CD271 enriched MSCs from BM, FH and RIA

**Fig 4:**
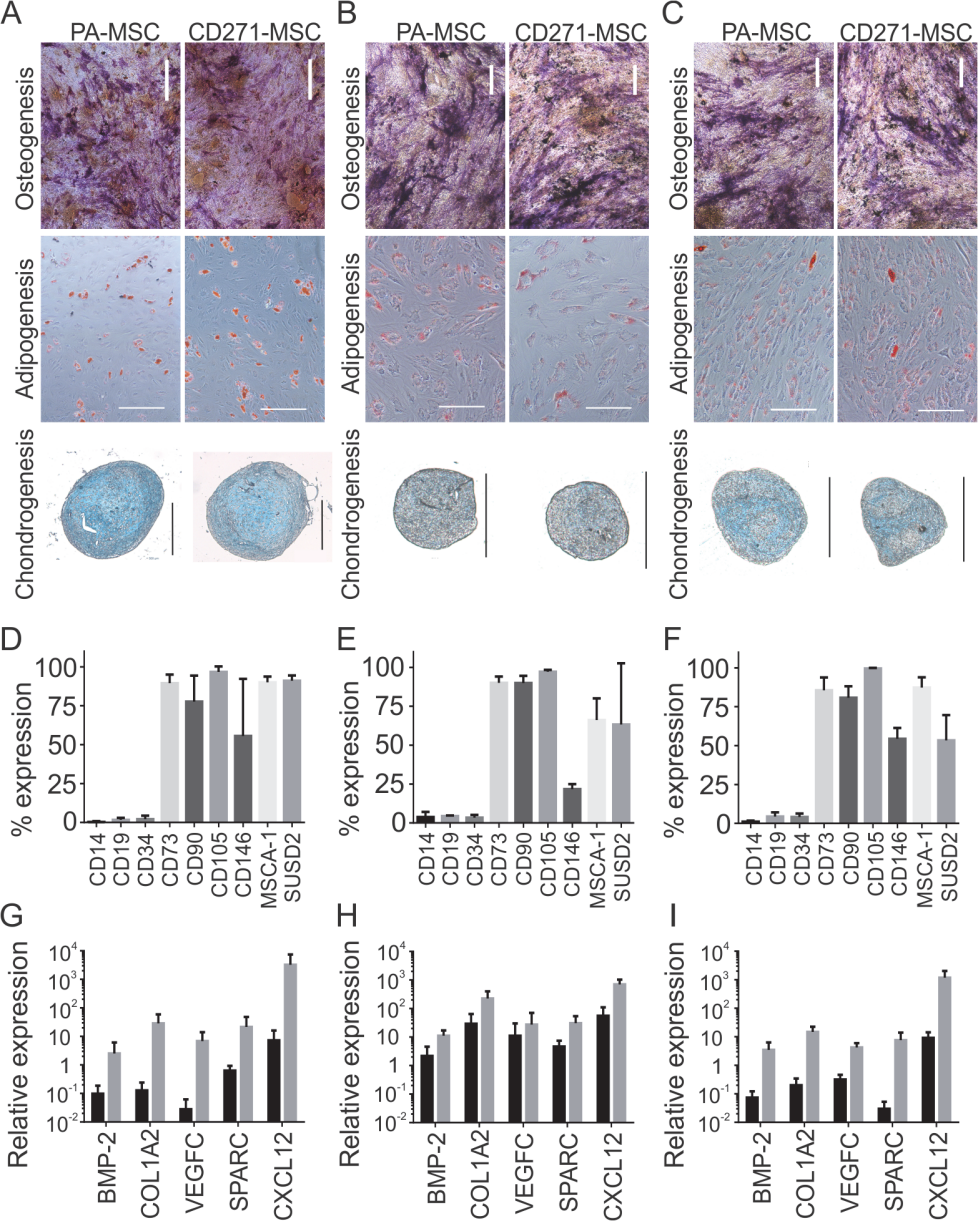
Functional analysis, phenotypic profile and transcripts expression of CD271 selected MSCs from BM, FH and RIA. Differentiation potential of PA-MSCs and CD271-MSCs. Osteogenesis, adipogenesis and chondrogenesis was measured on day-21 post-induction by staining with alkaline phosphatase/von Kossa, Oil Red O and Alcian Blue, respectively in cells selected from BM **(A),** FH **(B)** and RIA **(C)**, all size bars represent 500μm. Phenotypic analysis of the CD45^−/low^CD271^+^ population observed in BM **(D)**, FH **(E)** and RIA **(F)**. Analysis of the total expression of BMP-2, COL1A2, VEGFC, SPARC and CXCL12 transcripts relative to HPRT in BM **(G)**, FH **(H)** and RIA **(I)** pre (black bars) and post (grey bars) CD271 enrichment. (All data n = 3).

The functional and phenotypic characteristics of CD271 selected cells from BM, FH and RIA were compared next. In every case CD271 selection resulted in the isolation of cells capable of osteogenic, adipogenic and chondrogenic differentiation (Fig. 4A-C). Differentiation capacity was comparable to MSCs selected by plastic adherence as assessed by positive staining using alkaline phosphatase/von Kossa, Oil Red O and Alcian Blue respectively.

Analysis of the extended phenotype of CD45^−low^CD271^+^ MSCs revealed a high degree of similarity with regard to the expression of the classical phenotypic markers of MSCs. CD45^−low^ CD271^+^ cells in all tissues did not express CD14, CD19 and CD34 and had high expression of CD73, CD90 and CD105 (Fig. 4D-E). Two proposed alternative markers for MSC enrichment MSCA-1 and SUSD2 were highly expressed on CD45^−low^ CD271^+^ cells from the BM (mean of 90% and 91% respectively, Fig. 4D). However, in FH the proportion of cells expressing MSCA-1 and SUSD2 fell to 66.1 and 63.3, respectively (Fig. 4E). In RIA fluid MSCA-1 was highly expressed on CD45^−low^ CD271^+^ cells (87.4%), but this was not the case for SUSD2 (63.25%, Fig. 4F).

Altogether, these data confirmed that the selection of MSCs using clinical-grade CD271 microbeads resulted in the isolation of native MSCs with classical MSC functional and phenotypic characteristics.

### CD271 selected fractions express elevated levels of transcripts important in bone regeneration

Relative expression of BMP-2 transcript was increased (mean of 311-fold) by CD271 enrichment from BM (Fig. 4G). For VEGFC, an even higher level of enrichment (733-fold) was observed. COL1A2 and SPARC were also highly enriched (687- and 42-fold respectively) (Fig. 4G). Finally, the relative expression of CXCL12 was increased by the greatest degree following CD271 enrichment from BM (1381-fold, Fig. 4G). The expression of these transcripts were uniformly increased in FH although to a lesser degree (Fig. 4H), possibly reflecting much higher initial MSC content in this tissue, and therefore higher initial relative expression. Transcript levels in RIA fluid pre and post enrichment closely resembled those in BM (Fig. 4I).

In summary, CD271 selection led to substantially increased expression levels of BMP-2, VEGFC, COL1A2, SPARC and CXCL12 transcripts.

## Discussion

This study is the first assessment of a CD271-based GMP compliant immunomagnetic enrichment procedure to isolate uncultured MSCs for their use in bone repair applications. In every case the selection procedure resulted in a substantial increase in MSC purity and was accompanied by an increased expression of transcripts directly involved in bone formation and vascularisation. Overall, our data demonstrated the feasibility to successfully enrich functionally competent MSCs using an appropriate marker, appropriate device and appropriate procedure for clinical use. We also provided the first evidence supporting the possibility and feasibility to acquire clinically-relevant numbers of uncultured and functionally viable MSCs from discarded FHs or RIA fluids for their future autologous use.

We found that cultures initiated with CD271-selected cells were similar to plastic adherence selected cultures from the same donors, consistent with observations by Poloni *et al* [49] and extended these observations to CD271-selected MSCs from FHs and RIA fluids. Both plastic adherence and CD271 selected cultures possessed classical MSC phenotype [37] and were capable of tri-lineage differentiation *in vitro*. *In vivo* bone forming capacity of CD271 derived cultures has been demonstrated in previous studies [23,50].

Although we observed that cells expressing the CD45^-/low^CD271^+^ phenotype in all three tissues shared very similar expression levels of standard MSC markers such as CD73, CD105 and CD90 [37], we were aware that tissue residence (i.e. within trabecular or cortical bone) could have an additional impact on their *in vivo* composition [50–52]. To assess potential differences in MSCs selected from BM aspirates, FHs and RIA, we investigated the relative expression of two additional markers that have been suggested to offer highly specific selection for MSCs in BM, MSCA-1 and SUSD2 [38,39]. We confirmed the findings by the Buhring group that in BM aspirates MSCA-1 and SUSD2 were both uniformly and specifically expressed on CD45^−/low^CD271^+^ cells (i.e. were not expressed outside of the CD45^−/low^CD271^+^ population). However, in FH and RIA preparations, a significant proportion of CD45^−/low^CD271^+^ cells lacked expression of either MSCA-1 or SUSD2. It is therefore possible that in BM aspirates these markers may offer superior selectivity for MSCs by avoiding collection of the ‘passenger cell’ population however we believe that such MSCA-1 or SUSD2-based selection could in fact miss out some MSCs, if FHs or RIA fluids are used as a starting material. Regarding the enrichment of CD45^+^CD271^low^ ‘passenger’ cells in general, in all cases these were enriched to a lesser degree than CD45^−/low^CD73^+^ MSCs or CFU-Fs, suggesting that clinical-grade CD271 microbeads preferentially selected for MSCs, which express higher levels of CD271 [16,23]. The presence of other contaminating cells, which could potentially elicit an immune response, indicated that further improvements in this technology would be required to consider the use of such isolates in allogeneic settings.

In terms of autologous applications, it is the dose and volumetric concentration of MSCs, rather than their purity, appears to be critical. Hernigou et al showed that successful treatment of fracture non-union, by percutaneous injection of concentrated BM, was associated with an average total dose of 30,000 MSCs, measured by CFU-F assay at a concentration of ≥1000 CFU-Fs/ml [53]. The advantage of MSCs enriched using the CliniMACS system in this context is that the Post-fraction can be re-suspended in any final volume making concentrations of over 1000 CFU-Fs/ml easily attainable. Up to now the direct engraftment, proliferation and differentiation of MSCs injected into the fracture site has not been demonstrated however it is very plausible that injected cells exert their regenerative by the release of trophic factors [54,55], rather than extensive in situ expansion and differentiation. A possibility underlined by our findings showing a substantial increase in VEGF and SDF-1 post CD271 enrichment.

With over 71,000 total hip replacements performed annually in England and Wales alone [56], FHs offer an abundant readily available alternative to BM. However, the osteoarthritic environment in which these MSC reside may affect their functionality [57,58] and be a factor limiting their clinical allogeneic use. In comparison, reaming waste fluid represents a more attractive MSC source for future applications; it does not require enzymatic digestion for MSC extraction [33], and offers large volumes of easily obtainable, MSC-rich material that is currently discarded but could be used immediately or banked for the manufacture of high-purity MSC isolates. It must be noted that any MSC preparation is subject to patient related factors known to affect MSC potency such as donor age [8] and these should be taken into account when considering RIA-fluids for MSC therapy. Although the total MSC content following enrichment from RIA fluids was lower than anticipated (an average of 12,000 MSCs measured by CFU-F assay), we believe it can be significantly improved by further optimising fluid collection and tissue handling, including more rigorous anti-clotting measures.

In summary, the utility and commercial viability of MSC-based therapies for bone regeneration and in broader therapeutic settings, with or without culture expansion, are dependent on several factors the most salient of these are: safety, purity, cost and time required for processing (Table 2). The clinical uptake of novel therapeutic interventions based on culture-expanded MSCs is currently impeded by high regulatory demands and cost implications. CD271-based MSC selection from RIA waste fluids would significantly increase both MSC numbers and their purities and as such represents the most viable route for the clinical use of uncultured MSCs in the autologous settings.

**Table 2:** Summary of the advantages and disadvantages of uncultured MSCs for clinical applications.

Factor	Advantage	Disadvantage
Safety	Potential of spontaneous transformation and transfer of animal pathogens [9–11] is limited by avoiding *in vitro* culture.	Potential carry-over of undesirable phenotypic characteristics in case of FH [57,58].
Purity	Cells are not subject to *in vitro* aging associated with culture expansion [8,59], therefore no or minimal loss of native phenotypic [21,60] and functional characteristics.	Currently-achieved purity levels are unlikely to be sufficient for allogeneic use.
Cost	Reduced cost due to lower regulatory burden [12] and reduced use of GMP-grade culture facilities and reagents.	Total cost is dependent on the application and the numbers of MSCs required; these may be limited by the source material.
Time	Cells are ready for use within hours and do not require a lengthy expansion period.	For autologous use extraction/enrichment procedure must fit with intraoperative time frame.
